# How should we interrogate the hypothalamic-pituitary-adrenal axis in patients with suspected hypopituitarism?

**DOI:** 10.1186/s12902-016-0117-7

**Published:** 2016-06-17

**Authors:** Aoife Garrahy, Amar Agha

**Affiliations:** Division of Endocrinology, Beaumont Hospital, Dublin, Ireland; RCSI Medical School, Dublin, Ireland

## Abstract

Hypopituitarism is deficiency of one or more pituitary hormones, of which adrenocorticotrophic hormone (ACTH) deficiency is the most serious and potentially life-threatening. It may occur in isolation or, more commonly as part of more widespread pituitary failure. Diagnosis requires demonstration of subnormal cortisol rise in response to stimulation with hypoglycemia, glucagon, ACTH(1-24) or in the setting of acute illness. The choice of diagnostic test should be individualised for the patient and clinical scenario. A random cortisol and ACTH level may be adequate in making a diagnosis in an acutely ill patient with a suspected adrenal crisis e.g. pituitary apoplexy. Often however, dynamic assessment of cortisol reserve is needed. The cortisol response is both stimulus and assay- dependent and normative values should be derived locally. Results must be interpreted within clinical context and with understanding of potential pitfalls of the test used.

## Background

Hypopituitarism is a clinical syndrome of deficiencies in one or more pituitary hormones of which adrenocorticotrophic hormone (ACTH) deficiency resulting in adrenal failure is the most serious and potentially life-threatening feature. ACTH deficiency can present as a part of generalized pituitary failure (multiple pituitary hormone deficiency, MPHD) or less commonly as an isolated entity. In the most severe cases, it can manifest acutely and dramatically as a life-threatening adrenal crisis with vascular collapse especially during an intercurrent illness while in other cases, the features are more subtle and the onset is gradual. It may also be diagnosed during assessment of pituitary axes as part of routine practice in those with pituitary tumours or following pituitary surgery or radiation therapy in otherwise well patients with little or no symptoms.

### Physiology

ACTH is a 39 amino acid peptide produced in the anterior pituitary by proteolytic cleavage of the much larger precursor polypeptide proopiomelanocortin (POMC). POMC gene expression, processing to ACTH and ACTH secretion are stimulated by corticotropin releasing factor (CRF) which is secreted in the hypothalamus [1]. These processes are under negative feedback by glucocorticoids. ACTH secretion can be suppressed by exogenous glucocorticoids via this negative feedback mechanism and this represents the most common cause of ACTH deficiency.

ACTH acts on the G protein coupled melanocortin 2 receptor in the zona fasiculata of the adrenal cortex to stimulate the synthesis of cortisol. In addition to this rapid effect, ACTH induces steroidogenic gene transcription and causes adrenal hypertrophy leading to an increase in the long-term capacity of the adrenal gland to generate cortisol [2]. This results in the adrenal hypertrophy seen in chronic ACTH excess. In contrast, in chronic ACTH deficiency adrenal atrophy can occur. Cortisol circulates in the blood bound to cortisol binding globulin (CBG) and free cortisol binds to the glucocorticoid receptor in target tissues to regulate gene transcription as well as exerting rapid, non-genomic effects [1, 3].

ACTH also stimulates the production of adrenal androgens, primarily dehydroepiandrosterone (DHEA), from the zona reticularis. These represent the main source of circulating androgen in females. Mineralocorticoid production is spared in central hypoadrenalism as mineralocorticoid secretion is primarily mediated by the renin-angiotensin system [4].

### Clinical context

ACTH deficiency can be congenital or acquired due to structural or functional diseases of the pituitary or hypothalamus. It can occur in isolation or as part of more widespread pituitary failure. The most common cause of ACTH deficiency is ACTH suppression and subsequent adrenal atrophy due to chronic glucocorticoid use. Other causes are outlined in Table 1.

**Table 1:** Causes of central hypoadrenalism

Congenital	Acquired
Genetic	Tumor
* Isolated ACTH deficiency*	Non-functioning pituitary adenoma
POMC mutation/cleavage defect	Functional pituitary adenoma
Mutations in POMC transcription factors (TBX19)	Craniopharyngioma
	Pituitary metastases
* Associated with other pituitary deficiencies*	Germinoma
PROP1, LHX3, LHX4, HESX1, OTX2 mutations	Other tumours including astrocytoma, meningioma.
Midline Defects	Iatrogenic
Septo-optic dysplasia (without HESX1 mutation)	Exogenous glucocorticoids
	Pituitary surgery
	Cranial irradiation
	Post-treatment for hypercortisolism
	Opiates
	Infiltrative
	Neurosarcoidosis
	Histiocytosis X
	Haemochromatosis
	Inflammatory/Infective
	Hypophysitis (lymphocytic, granulomatous)
	Post-basal meningitis, abscesses, encephalitis.
	Traumatic/vascular
	Traumatic brain injury
	Subarachnoid haemorrhage
	Sheehan’s syndrome
	Miscellaneous
	Idiopathic
	Pituitary apoplexy
	Empty sella syndrome
	Rathkes cleft cyst

Interrogation of the hypothalamic pituitary axis may be required acutely or in a more routine setting. The clinical scenario can mandate which test is performed. For example a random cortisol and ACTH level may be adequate in making a diagnosis in an acutely ill patient with a suspected adrenal crisis e.g. pituitary apoplexy. In other situations a low morning cortisol level in an at risk patient may also be sufficient in diagnosing ACTH/cortisol deficiency. Often however, dynamic assessment of cortisol reserves is needed (see below).

In hypopituitarism, there is generally a specific sequential failure of pituitary hormones with GH being the most common pituitary hormone affected followed by gonadotrophins and culminating in loss of ACTH and thyroid stimulating hormone (TSH) [5]. ACTH however can be the first or only pituitary hormone affected in certain situations such as lymphocytic hypophysitis [6] or suppression of the hypothalamic-pituitary-adrenal (HPA) axis by exogenous glucocorticoids.

Detection and treatment of central hypoadrenalism is important as it has been shown to be associated with increased morbidity and mortality [7, 8].

### Testing for central hypoadrenalism

Interrogation of the HPA axis is performed as part of a formal screening process for hypopituitarism in patients with organic hypothalamic-pituitary disease such as those with sellar/parasellar tumours, post pituitary surgery or apoplexy, history of cranial irradiation or traumatic brain injury. In other situations the assessment may be triggered by suggestive symptoms such as fatigue, unexplained weight loss, spontaneous hypoglycaemia or hyponatraemia.

### Timing of assessment

#### ACTH suppression from exogenous steroids

ACTH suppression and subsequent cortisol deficiency can result from chronic glucocorticoid use, can be unpredictable regardless of dose and duration of glucocorticoid and recovery can take weeks to years [4, 9]. Patients on long-term glucocorticoids for inflammatory conditions are frequently referred to specialist endocrine services for assessment of possible ACTH suppression. As prednisone can cross-react with the cortisol assay we suggest waiting until the patient is tapered to a dose of 5 mg at which point they are switched to an equivalent dose hydrocortisone 10 mg twice daily and a dynamic test can be carried out with the dose held the afternoon before and morning of the test.

#### Pituitary surgery/apoplexy

All patients with sellar/parasellar tumours should be subjected to an assessment of HPA axis function, with perhaps the exception of those with pituitary microadenomas who are at a very low risk of ACTH deficiency. Patients should undergo post-operative interrogation of the HPA axis regardless of whether they had pre-operative ACTH deficiency as there is potential for these patients to gain functional recovery providing that viable normal pituitary tissue remains in situ [10].

ACTH deficiency is the most common deficit observed in pituitary apoplexy occurring in 50–80 % of cases [11]. Due to its high frequency and potentially life-threatening effects, empiric parenteral corticosteroid is administered, when possible preceeded by blood drawing for serum cortisol levels. Pituitary function can recover following surgical decompression [12, 13]. Therefore post-operative re-assessment of the HPA axis is required.

A 08.00 h plasma cortisol can be measured on day 1–3 post-operatively in patients not treated with glucocorticoids and day 3–5 in patients covered with glucocorticoids once the dose is tapered down to physiological replacement [14] with the second dose of hydrocortisone given early in the afternoon (if hydrocortisone is used) and the morning dose of hydrocortisone not given until after the sample is drawn. However, there are conflicting opinions regarding what constitutes a safe level of 08.00 h plasma cortisol [15–17]. Patients with a 08.00 h unstressed cortisol of >400 nmol/L have an extremely low risk of ACTH deficiency and no further dynamic testing of the HPA axis is required [18, 19]. Conversely, patients with a post-operative 08.00 h cortisol of <100 nmol/L are invariably ACTH deficient and glucocorticoid replacement should be commenced. Patients with a 08.00 h cortisol between these values require definitive testing [17].

The alternative, and our preferred, approach is to routinely perform a dynamic test of HPA axis function 6–8 weeks post-operatively. This has the advantage of facilitating early discharge of patients post-operatively on empiric glucocorticoid replacement rather than waiting for day 3–5 cortisol values and may better identify those patients with later recovery of cortisol secretion.

#### Traumatic brain injury

Hypopituitarism is a common occurrence among survivors of severe or moderate traumatic brain injury (TBI) with an estimated prevalence of 11–35 % among adult long term survivors [20]. Acute post TBI central hypoadrenalism is potentially life-threatening [21] and has shown to be associated with increased mortality [7]. In the acute post-traumatic phase morning cortisol measurements should be carried out on those with moderate (GCS 9-12) or severe TBI (GCS ≤ 8) or those with clinical features suggestive of ACTH deficiency ie. hypotension, hypoglycaemia or hyponatraemia. Patients should also be tested in the post-acute phase between 3–6 months after the event as early abnormalities can recover while new deficiencies may become apparent later [22].

#### Post cranial radiation

Hypopituitarism is a recognised consequence of cranial radiation both for pituitary and non-pituitary brain tumours. Its onset is dose and time dependent. The HPA axis seems to be the most radio-resistant site in patients who have undergone irradiation for non-pituitary disorders. Clinically apparent ACTH deficiency is uncommon (3 %) in patients receiving a total radiation dose of less than 50Gy to the hypothalamic-pituitary (HP) axis. The incidence dramatically increases in those receiving more intensive radiotherapy [23, 24] and is highest in those receiving pituitary radiotherapy for pituitary tumors [25]. It is our practice to routinely screen patients from 1 year post cranial radiation and annually thereafter unless a diagnosis of ACTH deficiency is made.

### Choice of test

The choice of the most appropriate test for the assessment of alterations in the HPA axis remains an area of considerable debate. The “ideal test” is one which is convenient, non-expensive, without side-effects while having a high degree of reproducibility and diagnostic sensitivity and specificity. Morning cortisol may be useful if it is clearly low or clearly healthy. In the appropriate clinical context, an early morning cortisol less than 100 nmol/L suggests a requirement for glucocorticoid replacement, while a level greater than 390–400 nmol/L strongly suggests an intact HPA axis [26, 27]. Most patients however require a dynamic assessment of the HPA axis (see Table 2)

**Table 2:** Evaluating the utility of the insulin stress test (ITT), glucagon stimulation test (GST) and the short synacthen (corticotropin) test (SST)

Test	Strengths	Drawbacks
Insulin tolerance test	1. Very high sensitivity 2. Assessment of ACTH and GH axes	1. Requires experience and medical supervision 2. Labour intensive and time consuming 3. Contraindicated in ischemic heart disease and seizure disorders 4. Hypoglycaemia not always achieved 5. Unpleasant for patients
Glucagon stimulation test	1. Assessment of ACTH and GH axes 2. Can be used in cases where ITT is contra-indicated	1. Nausea in up to 30 % cases 2. False positive (fail) rate 8 % 3. Time consuming
Short synacthen (corticotropin) test	1. Simple and well tolerated 2. Can be used in cases where ITT is contraindicated 3. Reliably excludes clinically significant ACTH deficiency	1. Does not assess GH axis 2. Unreliable if recent pituitary insult e.g. surgery, apoplexy 3. Theoretical concerns of false negative (pass) rate (when compared with ITT)

.

#### Insulin tolerance test

**Fig. 1:**
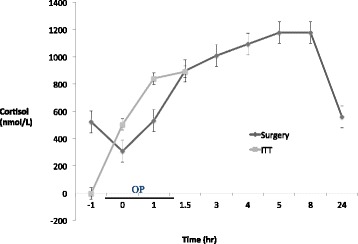
Cortisol responses to insulin and surgery in controls. Adapted with permission from Plumpton FS, Besser GM [28]

The insulin tolerance test (ITT) is regarded by many endocrinologists as the gold standard for interrogation of the hypothalamic pituitary axis. Hypoglycemic stress is a major stimulant of the HPA axis. In a seminal study in 1969, Plumpton and Besser showed that a normal cortisol response to ITT predicts an appropriate cortisol response during major surgery in both healthy and corticosteroid-treated patients [28]. Therefore normal response to ITT is highly reassuring (Fig. 1).

The ITT is performed under supervision by intravenous injection of 0.15 units/kg (0.1 units/kg if high suspicion of hypocortisolism, 0.2units/kg in insulin resistant states such as diabetes mellitus or acromegaly) soluble insulin with measurement of plasma cortisol at 0, 30, 45, 60, 90 and 120 min. Adequate hypoglycemia (blood glucose <2.2 mmol/L) with symptoms must be achieved to validate the test. The test has the advantage of also robustly assessing growth hormone (GH) production [29].

Contraindications to the ITT include history of seizures (particularly relevant in assessment of patients post TBI, subarachnoid haemorrhage (SAH) or with structural brain abnormalities who may have increased risk of seizures) and ischaemic heart disease (baseline ECG is required before performing test).

Some advocate against using the ITT in patients with a baseline cortisol of <100 nmol/L. However, Finucane et al showed the ITT to be safe in these patients and also an important diagnostic tool as 4 of 14 patients who had morning serum cortisol concentrations <100 nmol/L passed the ITT and remained well during long-term follow-up without glucocorticoid replacement [26]. Some centres recommend against the use of ITT in patients over age of 65 years due to safety concerns but in the previous paper none of the 9 % of patients who were over the age of 65 years had any adverse reactions to the test and we therefore do not use age as a contraindication to ITT in otherwise healthy individuals as long as the other exclusion criteria are taken into account.

#### Glucagon stimulation test

The glucagon stimulation test (GST) can be used to assess both the HPA and GH axes if the ITT is contraindicated, for example in those with heart disease. However the test is not usually used for the sole assessment of the HPA axis if GH assessment is not needed. Because of the false positive results, confirmatory tests (see below) are needed if the result is abnormal. 1 mg of glucagon (1.5 mg in those over 100 kgs) is given intramuscularly and samples taken for glucose, cortisol and GH at 0, 90, 120, 150 and 180 min. While the test is generally safe, up to 30 % of patients report nausea and occasional vomiting [30, 31]. Glucagon induced cortisol release has been shown to be ACTH dependent [32] but the precise mechanism of this physiological effect is not well-defined.

#### Short Synacthen (SST, corticotrophin)Test

Originally introduced to assess cortisol reserve in suspected primary adrenal failure (Addison’s disease), this test has also gained favour among many endocrinologists for the indirect assessment of the HPA axis [33–37] in cases of suspected secondary hypoadrenalism. Its diagnostic value relies on the assumption that chronic ACTH deficiency results in adrenal atrophy and therefore diminished response to exogenous acute ACTH stimulation [38]. The SST should not be used to assess for central hypoadrenalism therefore for at least 4–6 weeks post pituitary insult (e.g. surgery or apoplexy).

The high dose SST (HDSST) test involves the intravenous or intramuscular administration of 250 mcg of Synacthen (cosyntropin, ACTH (1-24)) a truncated ACTH peptide that has full biological activity but longer half-life than native ACTH (1-39). Baseline morning serum cortisol and ACTH (if central aetiology of the suspected hypoadrenalism is not clear) are taken and then cortisol measurement repeated 30 min after ACTH is administered. Another variant of the test is the administration of low dose (1 mcg) ACTH instead aiming for better sensitivity but comparing the performances of the two doses showed them to be equivalent [39]. Furthermore 1 mcg ACTH preparations are not available hence most centres use the high dose test.

#### CRF test

The CRF test has been proposed for the diagnosis of central adrenal insufficiency and for the distinction between secondary and tertiary adrenal insufficiency. CRF (100mcg) is injected intravenously and ACTH and cortisol measured every 15 min for 90 min. Patients with secondary adrenal failure have low ACTH levels that fail to respond to CRF while patients with tertiary (hypothalamic) failure show a prolonged and exaggerated rise in ACTH levels following CRF. The CRF test is not widely used to assess the HPA axis due to its unacceptably low sensitivity [40].

#### Overnight metyrapone test

The overnight metyrapone test assesses the negative feedback rather than stress related cortisol responses by utilizing the capacity of metyrapone to inhibit 11β-hydroxylase, the enzyme responsible for the conversion of 11-deoxycortisol to cortisol, thereby stimulating ACTH production and consequently increasing 11-deoxycortisol levels. While some studies have reported the metyrapone test to be reliable [41], other authors raised concerns about its sensitivity and specificity when using the traditional post-metyrapone 11-deoxycortisol level >200 nmol/L to define normality [42]. The diagnostic accuracy of the metyrapone test can be improved by integrating cortisol and ACTH levels with 11-deoxycortisol measurements [42]. Nevertheless, the assay for 11-deoxycortisol is a manual assay and is not available in most laboratories which limits the use of this test particularly as first line in the assessment of the HPA axis.

### Clinical and analytical considerations and caveats

As mentioned above, the SST should be avoided in the acute phase after a pituitary insult. The 30 min cortisol response to synacthen is the one which correlates with the peak cortisol response to hypoglycaemia [39]. The increment response to either the ITT or SST is less important than the absolute post-stimulation response which should be used to define normality.

Defining a normal cortisol response to hypoglycemia is a matter of contention. The study by Plumpton and Besser used a fluorimetric (Mattingly) method to measure serum cortisol and suggested a cut-off value of 580 nmol/L (20 ug/dl) for a normal response [28]. However, it is recognised that the fluorometric method reported a cortisol concentration that is 20 % higher than more modern immunoassays. More recent studies using contemporary cortisol assays redefined the normal cutoff for cortisol response to the ITT to closer to 500 nmol/L [43, 44] with one recent study suggesting a peak cortisol level of 414 nmol (14.8 ug/dl) to ITT to be the minimum acceptable cut-off value for healthy individuals [45]. In our institution we use a cut-off value of 500 nmol/L (18 ug/L) to define a normal response to ITT.

Normative cut-off values for peak cortisol in the GST are not easy to define but values between 450–500 nmol/l are appropriate. The GST is associated with an 8 % false positive (false fail) rate [31] especially when the baseline cortisol > 400 nmol/l.

Over the years, some endocrinologists have raised concerns that the SST may not be sensitive enough to diagnose central hypoadrenalism leading to missed diagnoses. This was based on studies comparing the cortisol response to hypoglycaemia with synacthen and finding that some patients who “failed” the ITT passed the SST. Unfortunately these studies have many limitations including failure to define normative cut-offs for ITT and or the SST locally in some studies (see below), issues regarding poor reproducibility with the ITT and use of the 60 min rather the 30 min response for the SST. In addition, it is well known that the ITT errs on the side of caution and probably overdiagnoses adrenal insufficiency as some patients do not show a normal cortisol response to hypoglycaemia but show a healthy response to major surgery [28].

However, studies looking at the outcome in patients who pass the SST and therefore were not given glucocorticoid replacement are reassuring. In a study of 148 hypothalamic-pituitary patients who passed the SST and were not treated with hydrocortisone, only 2 patients subsequently developed adrenal insufficiency, one with suspected evolving hypopituitarism and one who had a borderline response [36]. These results confirmed those of another similar study [37]. Therefore, when time-related clinical outcome is taken as the “gold standard” the SST rarely misses clinically significant adrenal insufficiency provided sufficient time has lapsed between the pituitary insult and testing [44].

For the SST, the cut-off for normality has been shown to be assay dependent, and therefore should be determined in each unit based on responses in healthy controls [46]. In our institution, we use 500 nmol/l as cutoff for normal cortisol response, with peak levels of 500–550 nmol/L interpreted to be safe for the purpose of withholding routine glucocorticoid therapy but these patients are prescribed stress dose glucocorticoids in times of intercurrent illness [36].

Dynamic tests assess ACTH/cortisol reserves rather than the adequacy of cortisol production from day-to-day under normal physiological conditions. Hence patients with partial or borderline responses may not need day-to-day glucocorticoid replacement as they have been shown to have normal cortisol production under unstressed physiological conditions [47]. As a precaution they should receive sick days glucocorticoid therapy [36].

Borderline results are often difficult to interpret. The ITT and the SST are not mutually exclusive and in some cases both tests can be used to rule in or rule out adrenal insufficiency when one test alone is borderline or when the test result does not support the clinical context. For example a patient with a pituitary tumour or after pituitary surgery with healthy morning cortisol level but a sub-optimal cortisol response to hypoglycaemia and otherwise normal anterior pituitary function is unlikely to have adrenal insufficiency and therefore a SST can be useful to verify or vice versa.

Analytical issues are also important when interpreting the result of a dynamic test. Reproducibility of the ITT has been shown to be 79 % compared with 88 and 83 % for the GST and the low dose ACTH stimulation test respectively [48]. Mean coefficient of variation of cortisol response to ITT is between 7–10 % and repeat cortisol values may differ by over 100 nmol/L [49–51]. No test correctly identifies all cases and borderline results should be interpreted in the clinical context. Failing to diagnose ACTH deficiency can have potentially life threatening consequences leading to adrenal crisis but over-zealous diagnosis can lead to unnecessary or over-treatment which has negative consequences for bone and metabolic health [52, 53].

Normative values should be derived locally as the cortisol response is both stimulus and assay-dependent. In one study by Clark et al, the 5^th^ percentile 30 min cortisol response to the SST ranged from 510 to 626 nmol/L depending on which of four different assays was used [46]. In a study of 129 patients comparing ITT, GST and low dose synacthen testing Simsek et al would have to treat 75, 65 and 40 % of the patients with glucocorticoids if they used the same cut-off of 500 mmol/L to diagnose central hypoadrenalism using these tests [48]. Furthermore, in clinical practice, laboratories measure total rather than the biologically active free cortisol. In situations where significant alterations in binding proteins exist, total cortisol measurement can be grossly misleading leading to misdiagnosis. For example septic patients have low levels of CBG and albumin hence their total cortisol measurement is much lower relative to their free cortisol measured by equilibrium dialysis [54] while patients taken estrogen will have an elevation in their total cortisol relative to free cortisol due to increased levels of binding proteins [55].

## Conclusion

The choice of test used to interrogate the HPA axis should be individualised for each patient and the results interpreted within clinical context and with understanding of potential pitfalls of the test used. An alternative test should be performed if the result is borderline and clinical suspicion is high. Clinical vigilance is required and patients should be made aware of the symptoms and signs of adrenal insufficiency so that assessment can be repeated if necessary. The results should be interpreted rationally and those with apparent borderline normal/abnormal results or values suggestive of partial ACTH deficiency could be treated with short courses of stress dose of glucocorticoids during incurrent illness only.

## Abbreviations

ACTH, adrenocorticotropic hormone; CBG, cortisol binding globulin; CRF, corticotropin releasing factor; DHEA, dehydroepandrosterone; ECG, electrocardiogram; GH, growth hormone; GST, glucagon stimulation test; HDSST, high dose short synacthen test; HP, hypothalamic pituitary; HPA, hypothalamic pituitary adrenal; ITT, insulin tolerance test; MPHD, multiple pituitary hormone deficiencies; POMC, proopiomelanocortin; SAH, subarachnoid haemorrhage; SST, short synacthen test; TBI, traumatic brain injury; TSH, thyroid stimulating hormone
